# Loss of Peroxiredoxin IV Protects Mice from Azoxymethane/Dextran Sulfate Sodium-Induced Colorectal Cancer Development

**DOI:** 10.3390/antiox12030677

**Published:** 2023-03-09

**Authors:** Pratik Thapa, Hong Jiang, Na Ding, Yanning Hao, Aziza Alshahrani, Eun Y. Lee, Junichi Fujii, Qiou Wei

**Affiliations:** 1 Department of Toxicology and Cancer Biology, University of Kentucky College of Medicine, Lexington, KY 40506, USA; 2 Department of Pathology and Laboratory Medicine, University of Kentucky College of Medicine, Lexington, KY 40506, USA; 3 Department of Biomolecular Function, Yamagata University, Yamagata 990-9585, Japan; 4 Markey Cancer Center, University of Kentucky College of Medicine, Lexington, KY 40536, USA

**Keywords:** peroxiredoxin, sulfiredoxin, colorectal cancer, redox, oxidative stress, tumorigenesis, tumor microenvironment

## Abstract

Peroxiredoxin IV (Prx4), a typical two-cysteine-containing member of the peroxidase family, functions as an antioxidant to maintain cellular redox homeostasis through the reduction of reactive oxygen species (ROS) via cycles of oxidation–reduction reactions. Under oxidative stress, all Prxs including Prx4 are inactivated as their catalytic cysteines undergo hyperoxidation, and hyperoxidized two-cysteine Prxs can be exclusively repaired and revitalized through the reduction cycle catalyzed by sulfiredoxin (Srx). Previously, we showed that Prx4 is a preferred substrate of Srx, and knockout of Srx in mice leads to resistance to azoxymethane/dextran sulfate sodium (AOM/DSS)-induced colon carcinogenesis. To further understand the significance of the Srx/Prx4 axis in colorectal cancer development, Prx4^−/−^ mice were established and subjected to standard AOM/DSS protocol. Compared with wildtype littermates, mice with Prx4^−/−^ genotype had significantly fewer and smaller tumors. Histopathological analysis revealed that loss of Prx4 leads to increased cell death through lipid peroxidation and lower infiltration of inflammatory cells in the knockout tumors compared to wildtype. Treatment with DSS alone also showed decreased infiltration of macrophages and lymphocytes in the colon of knockout mice, suggesting a role for Prx4 in inflammatory response. In addition, loss of Prx4 caused alterations in plasma cytokines and chemokines after DSS and AOM/DSS treatments. These findings suggest that loss of Prx4 protects mice from AOM/DSS-induced colon tumorigenesis. Thus, targeting Prx4 may provide novel strategies for colon cancer prevention and treatment.

## 1. Introduction

Colorectal cancer (CRC) is the third most common cancer and the third leading cause of cancer deaths in the US and worldwide [1]. Risks for CRC development in human include chronic inflammation (such as ulcerative colitis and Crohn’s disease), hereditary disorders (such as familial adenomatous polyposis and Lynch syndrome), and lifestyle related factors (such as obesity, and alcohol and tobacco usage). While progress has been made in the early detection of CRC in human, a significant amount of work remains to be performed to improve the long-term survival rate of patients. There is a high recurrence rate after endoscopic resection in some patient groups [2]. In addition, there is an increasing incidence of CRC in young adults below the age of 50 while mortality in elders also remains high [3]. Further understanding of the pathogenesis of CRC and development of more effective therapeutic strategies are in urgent need. 

Among all mechanisms of cancer development, oxidative stress has been widely recognized as one of the major cellular cues that contribute intrinsically to the occurrence of cell transformation, tumorigenesis, and promotion of and progression to malignancy. High levels of reactive oxygen species (ROS) and reactive nitrogen species (RNS) cause damage to DNA, lipids, and proteins by altering their structure and/or biological function [4]. Therefore, cells have evolutionarily developed various mechanisms of defense including expression of intracellular antioxidants to scavenge reactive species. Among them, peroxiredoxin IV (Prx4) is a member of the family of peroxidase that contains active thiol groups to reduce hydrogen peroxide, alkyl hydroperoxide, and peroxynitrite with high levels of selectivity and sensitivity [5,6]. Within the cell, Prx4 is found primarily in the endoplasmic reticulum (ER) where its peroxidase activity is essential for the maintenance of cellular redox balance and homeostasis. It also protects cells from ER stress which involves a process of oxidative protein folding through exchanging disulfide bonds with members of the protein disulfide isomerase family [7]. Under normal physiological conditions, the oxidation–reduction cycles of Prx4 and its substrate are accomplished through the coordination of thioredoxin/thioredoxin reductase or glutathione/glutaredoxin. Upon oxidative stress conditions, excessive ROS cause the hyperoxidation of the catalytic cysteine of Prxs, leading to the loss of enzymatic activity and even apoptotic cell death in normal cells [8]. However, cancer cells can survive oxidative stress through the upregulation of Prxs and/or expression of another redox enzyme, sulfiredoxin (Srx), which is exclusively dedicated to revitalizing hyperoxidized two-cysteine Prxs through an ATP-dependent oxidation–reduction cycle [9,10]. 

In our previous series of studies, we found that Srx preferentially interacts with Prx4 due to its intrinsically higher binding affinity, and the Srx–Prx4 axis contributes to the activation of oncogenic signaling pathways in different types of human cancer [9,11,12]. Unlike the ubiquitously expressed Prxs in normal tissues, Srx is not expressed in normal or cancer-adjacent normal colon tissues but is highly abundant in colorectal carcinomas. In mice, depletion of Srx does not generate any detrimental effect since Srx knockout (Srx^−/−^) mice are viable and have no defects in early development or adult life under laboratory conditions [13]. Interestingly, our previous studies showed that Srx^−/−^ mice are more resistant to tumorigenesis in either skin or colon carcinogenesis model induced by a complete protocol of carcinogen plus tumor promoter, i.e., 7,12-dimethylbenz[α]anthracene/12-O-tetradecanoylphorbol-13-acetate (DMBA/TPA) and azoxymethane/dextran sulfate sodium (AOM/DSS), respectively [14,15]. Therefore, the next logical question to ask is whether such effects of Srx are dependent on the presence of Prx4. To answer this, we established strains of Prx4-knockout (Prx4^−/−^) mice, and these mice were subjected to the AOM/DSS protocol to induce colorectal tumors. We found that Prx4^−/−^ mice developed fewer and smaller tumors than wildtype littermates. Series of strategies including pathohistological, immunochemical, and serological methods were used to determine how the loss of Prx4 affects the development of colorectal tumors in this model. Our mechanistic understanding of the Srx/Prx4 axis in tumorigenesis may provide novel insights to develop effective strategies for the prevention and treatment of colorectal cancer in patients.

## 2. Materials and Methods

### 2.1. Knockout Mice and Genotyping

All schemes of mouse breeding and experimental protocols were reviewed and approved by the University of Kentucky Institutional Animal Care and Use Committee (UK protocol 2016-2306). All procedures on mice were conducted following the Policy on Humane Care and Use of Laboratory Animals, and Guidelines of the Animal Care and Laboratory Animal Welfare (NIH). Mice, regardless of strain or genetic background, were all housed in standard cages in temperature-controlled environments under a 12 h light/12 h dark cycle with ad libitum access to standard chow (Teklad, Envigo, Indianapolis, IN, USA, cat no. 2918) and water unless otherwise indicated. Original Prx4^−/−^ mice in the C57BL/6 background were established by Iuchi et al. [16]. Prx4^−/−^ mice in the pure FVB/N background were further established by cross-breeding with FVB/N mice for multiple generations (>10).

For genotyping, genomic DNA was extracted from tail clips using genomic DNeasy Blood and Tissue Kit (Qiagen, Valencia, CA, USA, cat no. 69504). Briefly, a ~1 mm tip of the mouse tail was lysed using tissue lysis buffer and proteinase K at 56 °C overnight. After adding ethanol, the mixture was loaded into a spin column and washed twice; then, DNA was eluted with nuclease-free water. PCR-based genotyping was performed as reported previously [13]. The following primers were used at 10 μM each to amplify 1 μL target DNA using OneTaq 2× Master Mix (NEB, cat no. M0482): Prx4 wildtype-forward 5′–GAAATATCCTGGACATATGCTTTAAGA–3′; Prx4 wildtype-reverse, 5′–AAGATCCCCTGGAACAGAAGTTA–3′. This primer combination amplifies a 600 bp sequence upstream of the exon 1 of the Prx4 gene that is present in wildtype mice but not in Prx4^−/−^ mice. In addition, the following primer set was used to amplify disrupted intron 1 sequence (700 bp) in the targeting vector that is present in Prx4^−/−^ mice but not in wildtype mice: Prx4 KO-forward, 5′–ACTTCCGTTCCATGTTGAGC–3′; KO-reverse, 5′–ACAAACAAACCCAACCCTGA–3′. DNA was denatured at 95 °C for 5 min, followed by 30 cycles of 95 °C for 40 s, 58 °C for 30 s, 72 °C for 50 s, and a final extension at 72 °C for 2 min. The PCR product was loaded into 1% agarose gel with 6× gel loading dye and SYBR green, and then visualized using UV imager.

### 2.2. Mouse Tissue Extracts and Western Blotting

Fresh colon tumors or tissues collected from wildtype, and Prx4^−/−^ mice were lysed using radioimmunoprecipitation lysis buffer with the presence of protease inhibitor cocktail (Santa Cruz Biotechnology, Dallas, TX, USA, cat no. sc-24948). Protein concentration was measured using the detergent-compatible (DC) protein assay (BioRad, Hercules, CA, USA, cat no.5000116). Western blot was performed using a standard protocol. Briefly, proteins were electrophoresed on SurePage gel (GenScript, Piscataway, NJ, USA, cat no. M00654) and transferred to polyvinylidene difluoride membrane. Membranes were blocked for 1 h in 5% nonfat dry milk diluted in tris-buffered solution, incubated in primary antibody for overnight at 4 °C, washed multiple times, and then incubated in horseradish peroxidase-conjugated secondary antibody for 1 h. After further rinsing and washing, signals were detected using Amersham ECL Select Western Blotting Detection Reagent (Cytiva, Marlborough, MA, USA, cat no. RPN2235) and Amersham Imager 680 (GE Healthcare). Primary antibodies used were Prx4 1:10,000 (Abcam, Cambridge, UK, cat no. 184167) and β-actin 1:5000 (Sigma-Aldrich, St Louis, MO, USA, cat no. A2228). The specificity of anti-Prx4 antibody was validated and verified using knockout cell lines and mouse tissues as previously reported [17].

### 2.3. AOM/DSS Protocol, Tumor Measurement, and Histopathology Examination

The AOM/DSS protocol was performed as previously reported [18,19]. Briefly, mice at 8 weeks of age, including wildtype (*n* = 12) and Prx4-null (*n* = 14), were injected intraperitoneally with 10 mg/kg of azoxymethane (AOM, Sigma-Aldrich, cat no. A5486). Dextran sulfate sodium (DSS, Sigma-Aldrich, cat. no. D8906) was diluted to a concentration of 2% in autoclaved drinking water on the day of administration and supplied to mice 1 week and 8 weeks after AOM injection. At the 20th week after treatment, mice were humanely euthanized. Blood was collected in heparin-coated tubes and immediately centrifuged at 2000× *g* for 10 min at 4 °C; isolated plasma was stored at −80 °C. Mouse colon from the start of the anus were extracted and cleaned with phosphate-buffered saline. Tumor mass located within 1 cm of anus was counted as rectal tumors, and the remainder was counted as colon tumors. Tumor numbers were recorded, and dimensions were measured using a digital caliper. Tumor volume was calculated using the commonly accepted equation volume = (length × width^2^)/2 [20]. Tumor burden was obtained using the equation burden = (tumor area/total colon area), where area is the product of the length and the width [19]. After counting, samples of tumors were cut longitudinally and snap-frozen in liquid nitrogen to be stored at −80 °C or fixed in 4% paraformaldehyde and stored in 70% ethanol before proceeding with standard paraffin embedding, sectioning, and H&E staining. For histology and pathology assessment, serial sections of tumors from each genotype were obtained. The first of the sequential slides was stained with hematoxylin and eosin (H&E) and examined by the board-certified gastrointestinal pathologist to determine tumor histopathology. For the DSS-only protocol, two groups of mice at 8 weeks of age, including wildtype (*n* = 6) and Prx4-null (*n* = 6), were administered normal water or 2% DSS in autoclaved drinking water for 7 days and humanely euthanized; then, samples were collected and processed as described above.

### 2.4. Immunohistochemistry and Immunofluorescence

Immunohistochemistry (IHC) was performed using the Vectastain ABC-HRP Kit (Vector Laboratories, Burlingame, CA, USA, cat no. PK-6101, PK-6102) and 3,3′-diaminobenzidine (DAB) substrate kit (Vector Laboratories, cat no. SK-4100). Paraffin was first removed from tissue slides with two washes of xylene, and sections were immersed in decreasing concentrations of ethanol. Antigen retrieval was performed using citrate buffer pH 6.0 (Sigma, cat no. C9999). Slides were incubated in freshly prepared 3% hydrogen peroxide/methanol solution for 10 min to block endogenous peroxidase activity. Tissues were blocked with normal animal serum for 1 h and incubated with primary antibodies for 2 h at room temperature in a humidity chamber. Antibodies used were Prx4 1:500 (Abcam cat no. 184167), Ki67 1:40 (Abcam, cat no. 16667), 8-oxoG 1:100 (Santa Cruz, cat no. 130914), F4/80 1:40 (Cell Signaling, Danvers, MA, cat no. 70076S), CD86 1:100 (Santa Cruz, cat no. 28347), CD163 1:50 (Santa Cruz, cat no. 33715), CD138 1:100 (Santa Cruz, cat no. 12765), CD8 1:50 (Santa Cruz, cat no. 1177), CD4 1:100 (Cell Signaling, cat no. 90176T), CD19 1:100 (Cell Signaling, cat no. 25229T), PD-L1 1:100 (Cell Signaling, cat no. 13684S), PD-1 1:25 (Cell Signaling, cat no. 84651T). Samples were immersed in biotinylated secondary antibody for 1 h and avidin–biotinylated enzyme solution for 30 min. DAB was used as a chromogen, and hematoxylin was used for counterstaining. Images of slides were taken using Aperio ScanScope XT (Vista, CA, USA). Staining analysis was performed using HALO software (Indica Labs, Albuquerque, NM, USA). For immunofluorescence staining, slides were deparaffinized, and antigen retrieval was performed as described above. Tissues were co-stained for markers of inflammatory cells and Prx4 sequentially. Primary antibodies used were CD86 1:100 (Santa Cruz), CD163 1:50 (Santa Cruz), CD138 1:100 (Santa Cruz), and Prx4 1:500 (Abcam). Fluorophore-conjugated secondary antibodies used were Alexa Fluor 488 and Alexa Fluor 594 (Invitrogen, Waltham, MA, cat no. A11005, A11017). Tissue slides were mounted with ProLong Gold Antifade mountant with DAPI (Invitrogen, cat no. P36935). Images were captured using Cytation5 (BioTek, Winooski, VT, USA).

### 2.5. In Situ Apoptosis Assay

Mouse colon apoptosis assay was performed using terminal deoxynucleotidyl transferase-mediated dUTP nick end labeling (TUNEL) assay. The TACS2TdT-DAB in situ apoptosis detection kit was commercially obtained and assay was performed per manufacturer instructions (R&D Systems, Minneapolis, MN, USA, cat no. 4810-30-K). Briefly, deparaffinization and antigen retrieval were performed as described above. Tissues were incubated in Proteinase K for 30 min at room temperature followed by treatment with 3% hydrogen peroxide for 5 min. Tissues were covered with labeling reaction mix for 1 h at 37 °C, placed in streptavidin–HRP solution for 10 min, and immersed in DAB. Samples were then counterstained with hematoxylin, dehydrated, and mounted before visualization and analysis.

### 2.6. Tumor Lipid Peroxidation Measurement

Tumors isolated from mice colon were snap-frozen and embedded in the optimal cutting temperature medium (Sakura, Torrance, CA, USA, cat no. 4583). Cryosectioning was performed to obtain sections 5 μm in thickness (Leica Biosystems, Wetzlar, Germany, CM1860). Sections were stained with 2 μM C11 BODIPY 581/591 at 37 °C for 20 min (Invitrogen, cat no. D3861). Sections were fixed with paraformaldehyde for 10 min before mounting with ProLong Gold Antifade mountant with DAPI (Invitrogen). Images were taken using Cytation5 (BioTek).

### 2.7. Proteome Profiler Mouse Cytokine Array and Mouse Chemokine Array

The antibody-based array kits, which are capable of simultaneously measuring the levels of 25 chemokines and 111 cytokines in duplicates on the same membrane, were commercially obtained (R&D Systems, cat no. ARY020, ARY028). The membranes were first blocked for 1 h at room temperature. Next, 33.3 μL of plasma from three mice in each group was mixed. Membranes were incubated in the diluted 100 μL plasma sample, and antibodies were detected overnight at 4 °C. Streptavidin–HRP solution was applied for 30 min at room temperature, and the signal was detected using Amersham ECL Select Western Blotting Detection Reagent. Amersham Imager 680 was used to visualize the spots. The intensity of each spot representing the individual cytokine and chemokine was determined using ImageJ software. The relative spot intensity was obtained by normalizing with the intensity of the internal positive control on each membrane.

### 2.8. Statistical Analysis

Quantitative data are presented as means ± standard deviation (SD). Data were analyzed with Student’s *t*-test using GraphPad Prism 9. For calculation of the *p*-value, parameters of the two-tailed, 95% confidence interval were used for all analyses. A *p*-value less than 0.05 was considered statistically significant.

## 3. Results

### 3.1. Establishment of Prx4-Knockout Mice in FVB/N Background and Examination of Prx4 Expression in Intestinal Tract

**Figure 1:**
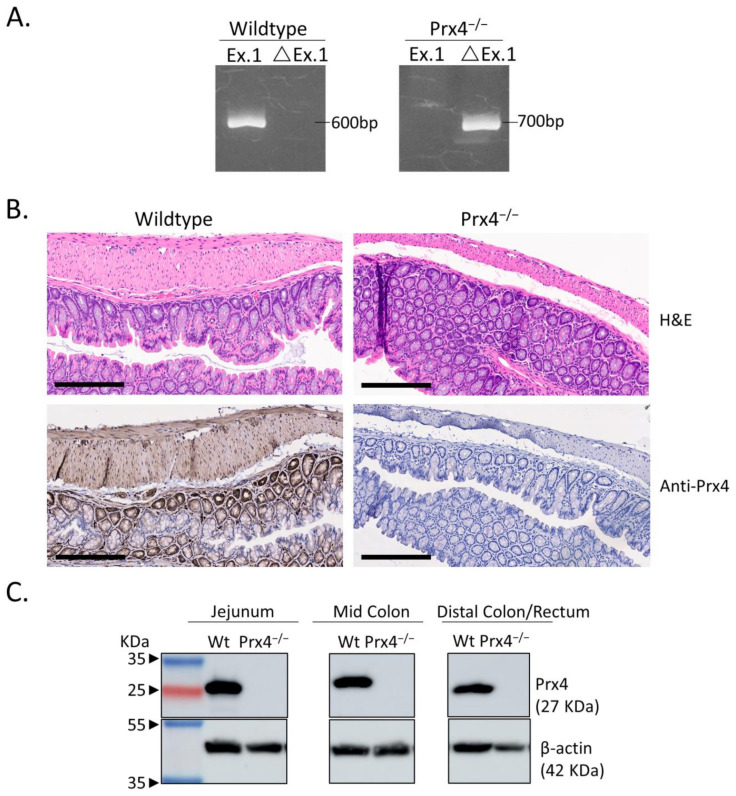
Genotyping of Prx4-null (Prx4^−/−^) FVB mice. (**A**) Genotyping was performed using a PCR-based amplification of Prx4 exon 1 sequence in genomic DNA of mouse tail. Ex.1 = exon 1 as expressed in wildtype mice and ΔEx.1 = deleted exon 1/shortened intron 1 in Prx4-knockout mice. (**B**) (Upper panel) Representative hematoxylin and eosin (H&E) staining of normal wildtype and Prx4-knockout mouse colon. (Lower panel) Immunohistochemistry (IHC) detection of Prx4 in wildtype and Prx4-knockout mouse colon. (**C**) Immunoblotting of Prx4 in wildtype and Prx4-knockout mice tissues jejunum, middle colon, and distal colon/rectum. Bar = 200 μm.

Prx4 whole-body knockout mice were originally established in the C57BL/6N background through deletion of exon 1 [16]. The targeting vector was constructed using Prx4 genomic DNA cloned from the b129/SVJ mouse genomic library; the neomycin cassette flanked by loxP sequences was inserted in intron 1, an additional loxP sequence was inserted upstream of exon 1, and the vector was transfected into embryonic stem cells. Following homologous recombination, embryonic stem cells were injected into blastocysts. Sperm collected from the resulting male mice was injected into unfertilized eggs and implanted into the uterus of pseudo-pregnant C57BL/6 female mice. The resulting Prx4^flox/+^ female mice were mated with male cre transgenic mice to generate Prx4^−/y^ male or Prx4^+/−^ female mice [16]. Similar to the normal phenotype of Srx whole-body knockout, loss of Prx4 in mice also does not cause any defects in both embryo development and adult life under laboratory conditions [13]. The susceptibility of mice to carcinogen-induced tumorigenesis varies due to differences in genetic background [21]. To minimize the complexity of data interpretation due to genetic variations in different mouse strains, we established Prx^−/−^ mice in the FVB/N background. All knockout mice were cross-bred with pure inbred, FVB/N wildtype for multiple generations (≥10) at the same breeding facility under the same feeding, drinking, and resting scheme. Regardless of strain background, we found that all Prx4-knockout mice were completely normal and fertile under laboratory conditions. The genomic loss of exons in Prx4 was verified by genotyping using PCR with a combination of target-specific primers (Figure 1A). Using a previously validated anti-Prx4 and IHC staining method [9,17], we examined the expression of Prx4 in the mouse intestinal tract. We found that Prx4-positive staining was ubiquitously present in the mucosa, submucosa, and muscularis layers of mouse colon. In particular, strong positive staining was mainly found in epithelial cells of the crypt in mouse colon (Figure 1B). Moreover, we examined the presence of Prx4 protein in the intestinal tract of wildtype mice by Western blotting. We found that Prx4 was present in the whole extracts of jejunum, colon, and rectum (Figure 1C). From these experiments, we found that Prx4 was ubiquitously present in the intestinal tract of wildtype mice, and that knockout of exon 1 led to the absence of Prx4 in Prx4^−/−^ mice.

### 3.2. Gross Tumor Analysis Reveals That Prx4^−/−^ Mice Are Resistant to AOM/DSS-Induced Colorectal Tumorigenesis

**Figure 2:**
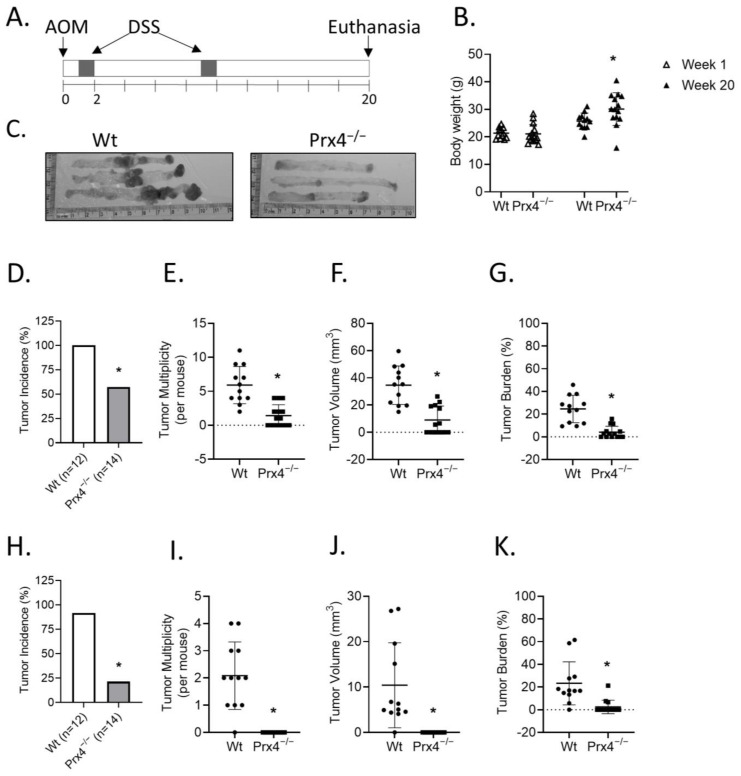
Prx4-knockout mice are resistant to AOM/DSS-induced carcinogenesis. (**A**) Schematic presentation of the AOM/DSS protocol. (**B**) Initial (week 1) and final (week 20) body weights of wildtype and Prx4-null mice. (**C**) Representative image of colons extracted from AOM/DSS treated mice. The average in Wt and Prx4-null mice of incidence, multiplicity, volume, and tumor burden percent in middle colon tumors (**D**–**G**) and distal colon rectum tumors (**H**–**K**), respectively. Compared with Wt group, * *p* < 0.05 (Student’s *t*-test).

To study the role of Prx4 in the development of colorectal tumor in vivo, we used a well-established AOM/DSS protocol to induce colitis-associated tumorigenesis in wildtype and knockout mice (Figure 2A). A total of 26 mice, including 12 wildtype (four male and eight female) and 14 Prx4-knockout (five male and nine female) were used in this study. All mice were initiated with a single dose of AOM through intraperitoneal injection, followed by two rounds of administration of DSS in drinking water, and euthanized 20 weeks after AOM injection (Supplementary Figure S1). Wildtype and Prx4-knockout mice had comparable average body weights before AOM injection, but Prx4-knockout mice had significantly higher weight than wildtype mice at the end of 20 weeks (Figure 2B). Loss of body weight with the development of AOM/DSS-induced tumor has previously been reported [22]. Visual examinations revealed that all colons extracted from wildtype mice had multiple, large tumors that were often aggregated to form a large mass in the middle colon, and the rectum was also obviously enlarged with tumors (representative image shown in Figure 2C, left panel). In contrast, colons extracted from Prx4^−/−^ mice bore fewer tumors with smaller size in the middle colon, along with a much-reduced size or absence of rectal tumors (representative images shown in Figure 2C, right panel). We counted the number and measured the size of all tumors formed both in the middle colon and in the rectum, and all data were quantitatively analyzed and compared for statistical significance between groups. Compared with wildtype, mice with Prx4^−/−^ showed significantly lower rates in colon tumor incidence (Figure 2D), multiplicity (Figure 2E), volume (Figure 2F), and burden (Figure 2G). Similar findings were also obtained when the incidence, multiplicity, volume, and burdens of rectal tumors were compared between wildtype and knockout mice (Figure 2H–K). These data indicates that loss of Prx4 alone is sufficient to cause resistance to AOM/DSS-induced colorectal tumorigenesis.

### 3.3. Prx4 Is Highly Expressed in AOM/DSS-Induced Colon Tumors, as Well as Infiltrated Macrophages and Plasma Cells, in Wildtype Mice

**Figure 3:**
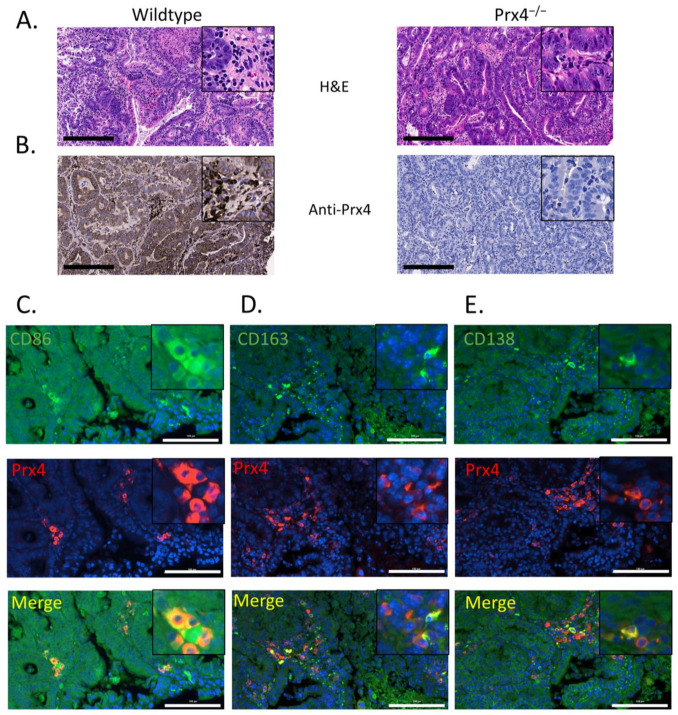
Prx4 is expressed in tumor-infiltrating immune cells. (**A**) Representative H&E staining of AOM/DSS-induced tumors extracted from Wt and Prx4^−/−^ mouse. (**B**) Representative IHC staining of Prx4 in Wt tumor showing high expression of Prx4 in tumor cells, as well as stromal cells, while Prx4 is not expressed in tumors from Prx4^−/−^ mice. Bar = 200 μm Double immunofluorescence staining of Prx4 with (**C**) M1 macrophage marker CD86, (**D**) M2 macrophage marker CD163, and (**E**) plasma cell marker CD138 in Wt tumors. Framed inserts indicate higher magnification. Bar = 100 μm.

To understand why the loss of Prx4 leads to resistance against colon tumorigenesis, we performed hematoxylin and eosin (H&E) staining and immunohistochemical examination of colon tumors extracted from wildtype and Prx4-null mice. H&E staining showed that wildtype tumors presented as poorly differentiated with disrupted aberrant crypts and abundant infiltration of inflammatory cells, while Prx4-null tumors were characterized as more differentiated crypts surrounded with fewer inflammatory cells (Figure 3A). The levels of Prx4 in wildtype tumors were also significantly increased compared to normal colon (Figure 3B and Figure 1B). In the IHC staining of Prx4 in wildtype tumors, we noticed that not only tumor cells but also some stromal cells in the tumor microenvironment had strong staining for Prx4 (Figure 3B). Bioinformatics analysis of RNA-sequencing data using the Gene Immunological Genome Project suggested that macrophages and plasma cells have high expression levels of Prx4 (Supplementary Figure S2) [23]. To confirm this in tumors, we performed double immunofluorescence staining for Prx4 plus markers of these cell types in tumors from wildtype mice. Macrophages can undergo a spectrum of activation states in response to different stimuli and are generally divided into two categories: M1-like macrophages that are involved in the proinflammatory response, and M2-like macrophages that are involved in the anti-inflammatory responses. In the context of the tumor microenvironment, M1 macrophages are considered antitumorigenic while M2 macrophages are considered immunosuppressive and protumorigenic [24]. We used antibodies against CD86 as a specific marker for M1 macrophages (Figure 3C) and against CD163 as a specific marker for M2 macrophages (Figure 3D). Tumor-infiltrating B lymphocytes play an important role in tumor malignancy and immunity as reported through their protumorigenic or antitumorigenic capabilities [25]. Plasma cells are terminally differentiated mature B lymphocytes. We used antibodies against CD138 as a marker for plasma cells (Figure 3E). The inflammatory cell markers CD86, CD163, and CD138 were stained with green fluorescence, while Prx4 was stained with red fluorescence in wildtype tumors (Figure 3C–E). We found that such co-staining frequently merged into yellow fluorescence, confirming that Prx4 is expressed in some macrophages and plasma cells (Figure 3C–E). Together, these data suggest that Prx4 is highly expressed in tumor cells, as well as tumor-infiltrating macrophages and plasma cells.

### 3.4. Loss of Prx4 Leads to Decreased Inflammatory Cell Infiltration into Tumors

**Figure 4:**
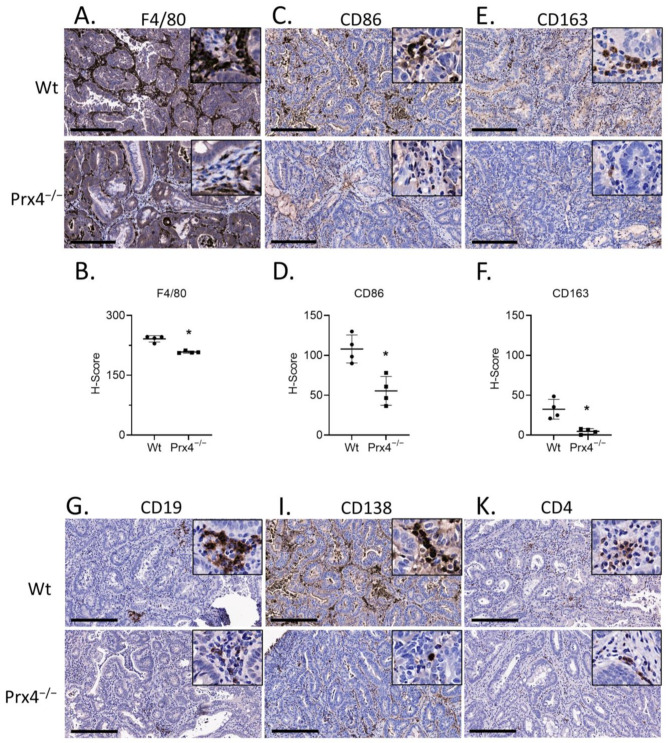

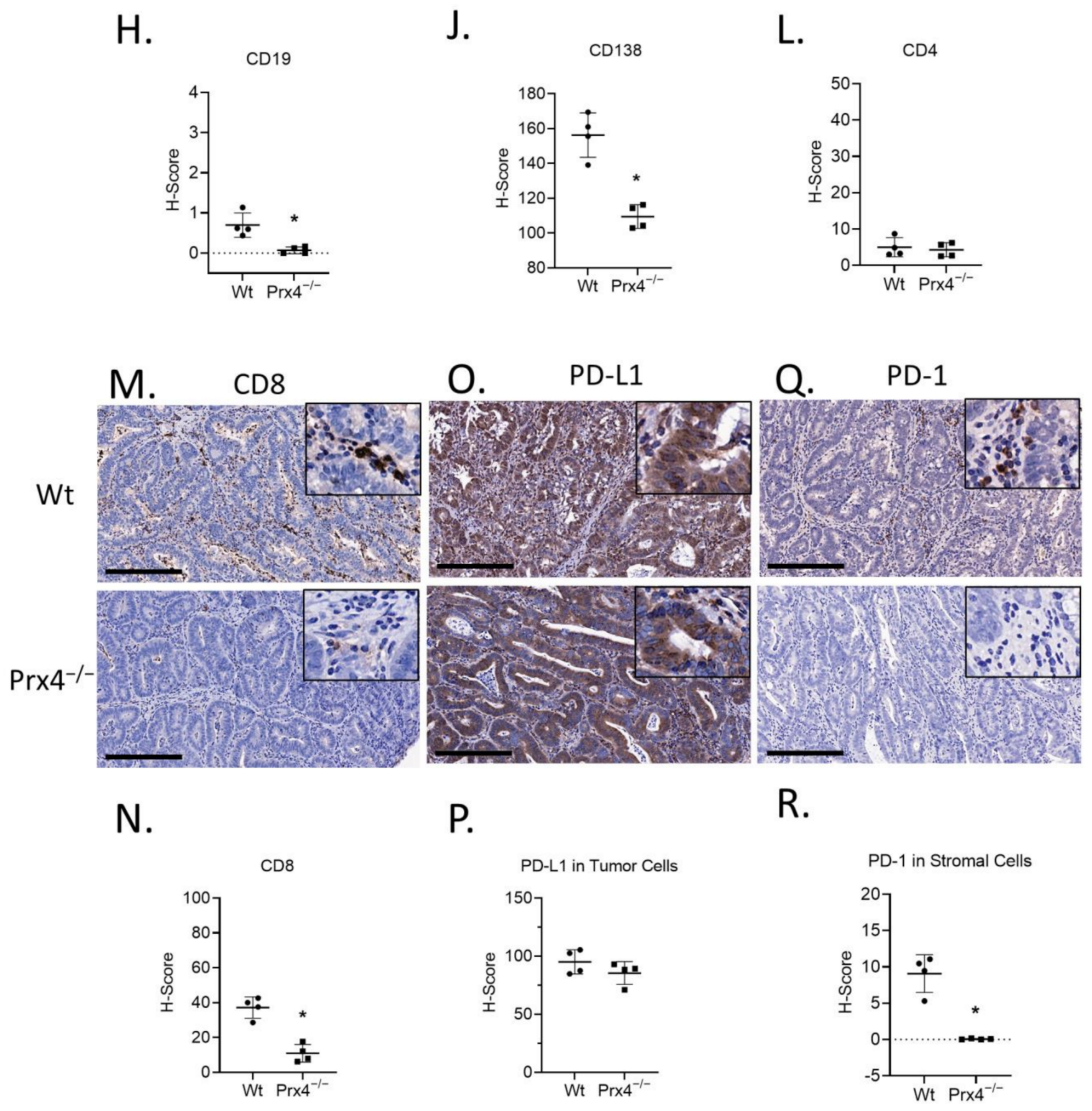
Prx4-knockout tumors have lower myeloid and lymphocyte infiltration compared to Wt. (**A**,**B**) Representative images of murine macrophage marker F/480 stained wildtype and Prx4-knockout tumors. H-score = histochemical score. (**C**,**D**) IHC of M1 macrophage marker CD86 showing lower staining in Prx4 KO tumor. (**E**,**F**) IHC of M2 macrophage marker CD163 showing lower staining in Prx4 KO tumor compared to Wt. (**G**,**H**) Decreased naïve B cell infiltration in Prx4 KO tumors as indicated by staining of CD19. (**I**,**J**) IHC of CD138 indicating lower infiltration of differentiated plasma cells in Prx4 KO tumors. (**K**,**L**) Similar levels of CD4+ staining were found between Wt and Prx4 KO tumors. (**M**,**N**) IHC staining of CD8 showing decreased recruitment of cytotoxic T cells in Prx4 KO tumors. (**O**,**P**) IHC of PD-L1 in tumor sections showing comparable staining. (**Q**,**R**) IHC detection of PD-1 in wildtype and Prx4 KO tumor sections. Compared with Wt group, * *p* < 0.05 (Student’s *t*-test). Bar = 200 μm.

Inflammation is known to contribute to tumorigenesis [26]. Since we observed strong expression of Prx4 in tumor-infiltrating immune cells, we speculated that the decrease in tumor burden in Prx4-knockout group was linked to decrease in inflammation. Therefore, we next examined immune cell infiltration in tumors of wildtype and Prx4^−/−^ mice. Macrophages are among the most abundant immune cells in the tumor microenvironment [27]. Using the AOM/DSS-induced colon tumorigenesis model, we previously found significantly decreased macrophage infiltration in tumors of Srx^−/−^ mice compared to those of wildtype [14]. To examine whether this was also true in Prx4^−/−^ tumors, we stained tumor slides with specific antibody to macrophage marker F4/80. Indeed, there were significantly fewer F4/80 positive cells in tumors from Prx4-null mice than those from wildtype mice (Figure 4A,B). We also used antibodies against CD86 as a specific marker for M1 macrophages (Figure 4C,D) and against CD163 as a specific marker for M2 macrophages (Figure 4E,F). We found that the presence of both M1 and M2 macrophages is significantly lower in Prx4^−/−^ tumors compared to wildtype tumors. 

In addition to macrophages, we compared the presence of lymphocytes in AOM/DSS-induced colon tumors. The markers used for B lymphocytes were CD19 for naïve B cells (Figure 4G,H) and CD138 for terminally differentiated plasma cells (Figure 4I,J). We detected lower presence of both cell types in Prx4-knockout tumors compared to those of wildtype. T cells are another key component of the colorectal tumor microenvironment and essential for immunotherapy [28]. T cells are broadly classified into two groups on the basis of the lineage markers CD4 and CD8. CD4^+^ T cells (helper T cells) secrete cytokines to enhance or suppress the proinflammatory response [29]. CD8^+^ T cells (cytotoxic T cells) can kill pathogen-infected or malignant cells [30]. We used these cell surface markers to stain the tumors and found that there was no significant difference in the infiltration of CD4^+^ cells between the groups (Figure 4K,L). However, CD8^+^ staining was significantly lower in Prx4-knockout tumors than in wildtype (Figure 4M,N). Together, these data indicate that loss of Prx4 decreases infiltration of macrophages, B cells, and cytotoxic T cells into the tumor microenvironment.

Programmed cell death protein 1 (PD-1) is a transmembrane protein expressed in T cells, B cells, monocytes, and dendritic cells [31]. Programmed death ligand 1 (PD-L1) is a transmembrane protein, expressed in immune cells, as well as a variety of nonhematopoietic cells, that binds to PD-1. PD-L1 ligation with PD-1 is important for immune homeostasis and to prevent autoimmunity. However, tumor cells also upregulate PD-L1 to escape immune surveillance [32]. Since we observed a decrease in immune cell population in the Prx4^−/−^ tumors, we next asked if PD-1 and PD-L1 expression was also affected in the tumors. PD-L1 was found to be highly expressed in tumor cells, and its levels were similar between the groups (Figure 4O,P). In contrast, positive staining of PD-1 was mainly found in infiltrated inflammatory cells from tumors of wildtype mice, while significantly reduced number and levels of PD-1 were observed in tumors from Prx4-null mice (Figure 4Q,R). This observation is consistent with the decrease in immune cell infiltration in Prx4-null tumors. Taken together, these data indicate that the absence of Prx4 leads to significant changes in the tumor microenvironment, characterized by reductions in macrophages, T lymphocytes, and plasma cell infiltration.

### 3.5. Loss of Prx4 Leads to Increased Rates of Apoptosis and Lipid Oxidation

**Figure 5:**
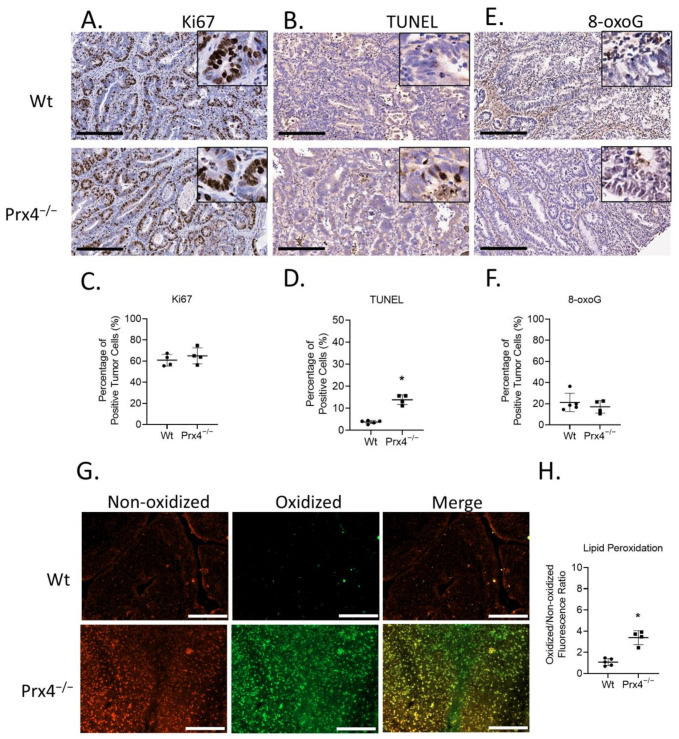
Prx4 knockout tumors have higher apoptosis and lipid peroxidation than Wt. (**A**,**B**) Similar levels of Ki67 staining were found between Wt and Prx4-knockout tumors. (**C**,**D**) TUNEL assay indicating increased rate of apoptosis in Prx4 KO tumors. (**E**,**F**) Nuclear 8-oxoguanine, a marker of oxidative DNA damage, stained at similar levels in the Wt, Prx4 KO groups. Bar = 200 μm. (**G**,**H**) C11- BODIPY 581/591 staining of tumor sections to measure lipid peroxidation. Bar = 100 μm. Compared with Wt group, * *p* < 0.05 (Student’s *t*-test).

We also examined tumors for markers of cell proliferation and cell death. Nuclear protein Ki67 was used as a marker for cell proliferation. There was no significant difference in the percentage of Ki67-positive tumor cells between the groups (Figure 5A,B). The terminal deoxynucleotidyl transferase-mediated dUTP nick end labeling (TUNEL) assay, which detects DNA fragmentation, was used to measure apoptosis in mouse tumors. TUNEL-positive cells were significantly increased in tumors from Prx4^−/−^ mice than those from wildtype mice (Figure 5C,D). This suggests that the lower tumor burden in the Prx4-knockout group is a consequence of increased apoptosis in Prx4^−/−^ tumors. Because Prx4 functions as scavenger of hydrogen peroxides, alkyl hydroperoxide, and peroxynitrite and protects cells from oxidative stress, we next asked if loss of Prx4 led to higher oxidative stress in the tumors. Oxidative DNA lesion 8-oxoguanine (8-oxoG) was used as a marker of oxidative DNA damage. We found that 8-oxoG immunoreactivity was not significantly different between the groups (Figure 5E,F). Since Prx4 is primarily distributed in the endoplasmic reticulum and extracellular matrix, and since Prx1 and Prx2 are still present in the nucleus, it is possible that loss of Prx4 does not affect DNA damage. To examine lipid peroxidation, snap-frozen tumors were cryosectioned and stained with BODIPY 581/591 C11, a fluorescent fatty acid analog, before fixation. In this assay, there is a shift of the fluorescence emission peak from ~590 nm (red fluorescence) to ~510 nm (green fluorescence) upon oxidation of the polyunsaturated butadienyl portion of the dye. Fluorescence microscopy examination of the stained tumors showed that Prx4 knockout tumors had significantly higher green fluorescence, indicating higher lipid peroxidation in Prx4 knockout tumors compared to wildtype (Figure 5G,H). Thus, loss of Prx4 did not increase oxidative DNA damage but did increase lipid peroxidation and apoptosis in Prx4^−/−^ tumors.

### 3.6. Loss of Prx4 Disrupts Cytokine-Mediated Signaling

**Figure 6:**
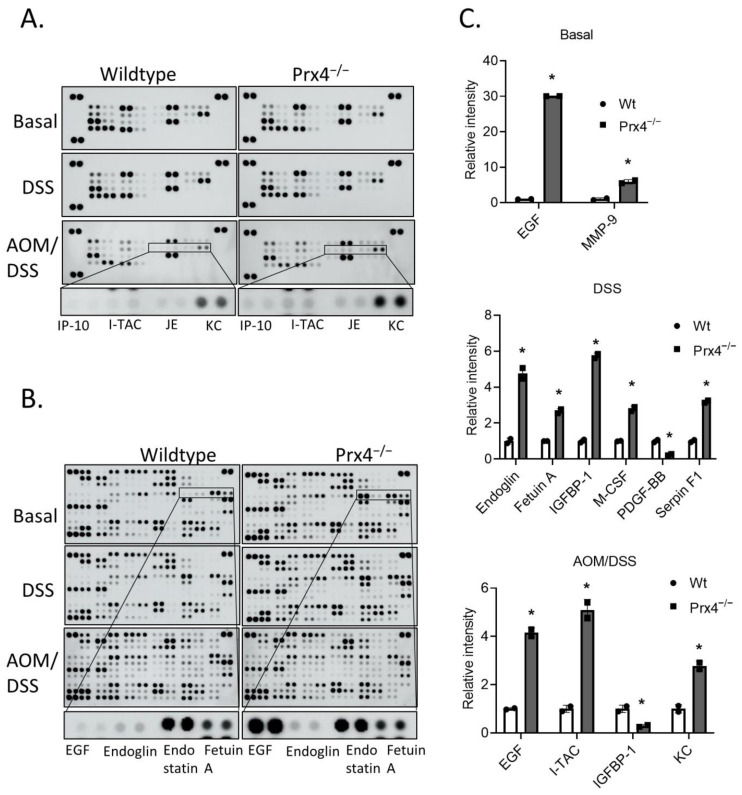
Loss of Prx4 affects chemokine- and cytokine-mediated signaling. (**A**,**B**) Proteome profiler mouse chemokine (**A**) and cytokine (**B**) array original blots using plasma isolated from basal (no treatment), DSS, and AOM/DSS treatment groups. Representative spots are highlighted. (**C**) Quantification of intensity of cytokine and chemokine duplicate spots identifying significantly different proteins under basal conditions (top panel), after DSS treatment (middle panel) and after AOM/DSS treatment (lower panel). Compared with Wt group, * *p* < 0.05 (Student’s *t*-test).

To understand the mechanisms of how Prx4 might regulate inflammation, we next treated wildtype and Prx4-knockout mice with 2% DSS for 7 days. We characterized the circulating cytokine and chemokine levels of wildtype and Prx4^−/−^ groups using proteome profiler array (R&D Systems). The layouts of arrays and names of chemokines and cytokines are shown in Supplementary Figure S3. For each group, plasma was collected after no treatment (basal), DSS treatment, or AOM/DSS treatment. Plasma from three mice in each group were mixed and incubated in membranes containing capture antibodies. Array signals were imaged and quantified using ImageJ. We found that loss of Prx4 affected secretion of a variety of proinflammatory and anti-inflammatory cytokines, as indicated by the difference in spot size and intensity (Figure 6A,B). Proteins with significant fold change compared to corresponding wildtype group are shown in Figure 6C. Specifically, untreated Prx4^−/−^ mice had higher levels of epidermal growth factor (EGF) and matrix metallopeptidase 9 (MMP-9) than untreated wildtype mice. Endoglin, Fetuin A, insulin-like growth factor-binding protein 1 (IGFBP-1), macrophage colony-stimulating factor (M-CSF), and Serpin F1 were upregulated in the Prx4-knockout group after DSS compared to wildtype, while platelet-derived growth factor (PDGF)-BB was downregulated. Lastly, at the end of AOM/DSS treatment, interferon-inducible T-cell alpha chemoattractant (I-TAC), keratinocyte-derived chemokine (KC), and EGF were higher, and IGFBP-1 was lower in the Prx4^−/−^ group relative to wildtype. Thus, these data suggest that Prx4 plays a critical role in regulating inflammation via cytokines. In addition to plasma, colon tissues from DSS-treated mice were also collected, fixed, and stained for different markers of immune cells as described above. Consistent with AOM/DSS treatment, we found that loss of Prx4 reduced the infiltration of CD86^+^ M1 macrophages, CD163^+^ M2 macrophages, CD138^+^ plasma cells, CD4^+^ T cells, and CD8^+^ T cells in Prx4^−/−^ colon (Supplementary Figure S4). Thus, Prx4 protects mice against DSS-induced inflammation by modulating cytokines and chemokines.

## 4. Discussion

It has been well recognized that strains of inbred mice do not bear the same genetics due to polymorphisms and multiple layers of regulatory elements. Such distinctions in genetics lead to significant variations in susceptibility to tumorigenesis among different mouse strains even exposed to the same tightly controlled protocol of carcinogen(s) [21,33]. Among them, FVB/N mouse has been reported to be highly sensitive to carcinogen-induced cancer development, such as DMBA/TPA-induced skin cancer AOM/DSS-induced colon cancer, and urethane-induced lung cancer [34,35,36]. Previously, we showed that Srx^−/−^ mice are resistant to AOM/DSS-induced colon tumorigenesis, identified that Prx4 is the major downstream substrate of Srx, and revealed that the integrity of the Srx/Prx4 axis is required for cancer malignancy [9,11,14].

In the present study, we aimed to identify the function of Prx4 in colon carcinogenesis. The AOM/DSS model was again used to induce colon cancer development in wildtype and Prx4-null mice in the FVB/N background. We demonstrated that the absence of this gene provided resistance to chemically induced tumor formation. The Prx4^−/−^ group had lower tumor incidence, multiplicity, volume, and tumor burden than wildtype mice. In mechanistic studies, we found that Prx4 knockout led to higher intratumoral cell death likely due to increased oxidative stress. The knockout of Prx4 also decreased infiltration of inflammatory cells into the tumor microenvironment and resulted in downregulation of PD-1 in tumor stroma. 

Our results presented here are in accordance with several previously published studies that examined the role of Prx4 in inflammation and cancer. It has been reported that Prx4 can activate NF-κB, a master regulator of inflammation, in T cells and macrophages [37,38]. We previously demonstrated that Srx contributes to tumorigenesis in chemically induced models of colon cancer and skin cancer [14,15]. Srx-null tumors in both studies had comparable cell proliferation but significantly higher apoptosis than wildtype tumors. In addition, loss of Srx reduced macrophage infiltration in colon tumors. Similarly, in urethane-induced lung cancer, Prx4 promoted chemically induced lung tumorigenesis. Human Prx4-expressing transgenic mice had more tumors than non-transgenic control mice [39]. The tumors in transgenic groups also had lower oxidative stress and higher macrophage infiltration. Furthermore, Prx4 has been suggested to promote progression of prostate cancer, pancreatic cancer, hepatocellular carcinoma and colorectal cancer [17,40,41,42]. Lastly, loss of Prx4 providing resistance to colitis fits the general trend observed when antioxidant enzymes are depleted in colitis models: loss of Prx1, Prx2, and Prx6 and the combined loss of GPx1 and catalase all improved colitis in mouse and rat models [43,44,45,46]. Appropriately increased oxidative environment upon silencing of antioxidant enzymes appears to increase expression of regulatory T cells and suppress inflammatory response. Exceptions to this trend include GPx2-knockout and Gpx1 and GPx2 double-knockout mouse models, which highlight the protective role of selenium [47,48]. Thus, our study further confirms the proinflammatory and oncogenic role of Prx4 in colorectal cancer. According to our findings that Prx4 promotes inflammatory tumor microenvironment, the next question we need to ask is whether myeloid cell-specific or lymphoid cell-specific knockout of Prx4 is enough to replicate results of whole-body knockout in an in vivo colon carcinogenesis model. In addition, it would also be interesting to investigate the consequence of double knockout of Srx and Prx4 on carcinogenesis resistance. With Prx4 being the primary substrate of Srx, it is likely that loss of either of these genes would yield identical results as combined loss. However, it is also possible the combined depletion would confer additive or synergistic resistance, thereby providing a new therapeutic approach. Therefore, future studies of their role in colitis and colitis-associated colorectal cancer are justified.

Prx4 loss caused upregulation of several proinflammatory and anti-inflammatory cytokines after DSS and AOM/DSS treatment. As mentioned above, Prx4 modulates NF-κB signaling, and many of the cytokines differentially expressed in our study are known targets of this transcription factor [49,50,51]. We observed increased plasma EGF and MMP-9 in Prx4KO mice compared to wildtype. The reason for this upregulation is not clear. We previously showed that loss of Srx contributes to reduction of EGFR signaling and MMP-9 protein expression [9,11]. It is possible that loss of Prx4 has a similar effect on these proteins and signaling pathways, resulting in a feedback loop which causes high circulation levels for compensation. Further studies are necessary to address the function of these upregulations in normal physiology. Among the cytokines differentially expressed in Prx4KO mice upon DSS treatment, increased Endoglin and Fetuin A and decreased PDGF-BB have previously been associated with reduced inflammation of the colon. Endoglin heterozygous mice were more sensitive to DSS treatment compared to wildtype mice, resulting in higher VEGF levels and angiogenesis [52]. In addition, in human subjects, increased serum Fetuin A is inversely associated with inflammatory bowel disease (IBD), suggesting a protective role for this protein [53]. IBD patients have also been reported to have higher plasma PDGF-BB compared to healthy controls [54]. However, even though Prx4 deficiency is protective overall against colitis, there was upregulation of proteins positively associated with gastrointestinal inflammation, namely, Serpin F1, M-CSF, and IGFBP-1 [55,56,57]. Targeting these proteins along with Prx4 could further strengthen resistance against colitis and tumor formation. 

After AOM/DSS treatment, plasma EGF was significantly higher in the Prx4^−/−^ group, although the difference between wildtype and Prx4^−/−^ plasma was reduced compared to basal conditions. Plasma EGF is significantly higher in cancer patients than in those with benign colorectal conditions [58]. In addition, IGFBP-1, which was reduced in Prx4KO mice, is inversely associated with colorectal cancer in human subjects [59,60]. High KC mRNA is also inversely associated with overall survival of stage IV patients (but there were no correlations to survival in stage II and III patients) [61]. Therefore, whether these cytokines have protumor or antitumor functions in Prx4KO mice is not clear. It might be worthwhile to conduct studies targeting one or more of these proteins along with Prx4 depletion to shed more light on their combined contribution to colon carcinogenesis. Meanwhile, I-TAC, which was upregulated in the Prx4^−/−^ group, is reported to have a protective role against colon cancer. Bioinformatics analysis showed I-TAC transcript is higher in stages I and II of CRC compared to stages III and IV, and that high I-TAC is associated with better overall survival of patients [62,63]. Thus, upregulation of these chemokines at least partially contributed to reduced colon tumor formation in Prx4KO mice.

Prx4 is upregulated in human IBD, and it has been suggested as a potential diagnostic marker for IBD [64]. Another Prx family member, Prx1, has also been associated with increasing severity of colitis [65]. However, whether Prx4 contributes directly to IBD progression or is a secondary response to altered intestinal microenvironment is not fully understood. Our results indicate that Prx4 promotes DSS-induced inflammation. However, these findings contrast with other data reporting an anti-inflammatory role of Prx4 after DSS [66]. The most notable discrepancy between this study and a previous report was in the type of mouse strains used. Specifically, Takagi et al. utilized male mice of the C57BL/6 background, whereas this study utilized both male and female mice of the FVB/N background. Different strains of mice have different responsiveness to DSS [67]. In addition to genetic differences between the mice, the differences in the mouse microbiome, as a result of housing in different institutions, also likely contributed to the inconsistency [68]. It remains critical for future studies to evaluate the effects of these factors on the function of Prx4.

Our study of colon carcinogenesis utilized the AOM/DSS model. After treatment, CRC develops from aberrant crypt foci and adenoma, mimicking the sequence of CRC formation in human [69]. However, AOM/DSS is ideally used to model nonhereditary, inflammation-driven CRC. Therefore, our findings apply best to colitis-associated cancer, and caution must be taken when translating the findings to other subtypes of CRC. Further studies must be conducted in other mouse models, such as mouse with mutations in the *Apc* gene, to investigate the function of Prx4 in development of familial colon cancer. Another limitation of our study is that the effect of Prx4 loss on host microbiota was not studied. Intestinal bacteria play an important role not only in metabolic activation of pro-carcinogen AOM, but also in increasing the severity of colitis upon DSS-induced epithelial injury [70,71,72]. Therefore, it is critical that future studies address how Prx4 modulates the intestinal microbiota at basal conditions and regulates response to infection. 

Overall, our results show an important role of antioxidant Prx4 in regulating colon carcinogenesis. Bioinformatics analysis of RNA-Sequencing datasets available in TCGA database showed that Prx4 transcripts are upregulated in both colon adenocarcinoma and rectum adenocarcinoma patient samples [17]. Our data presented here indicate a protumorigenic function of this upregulation. Knockdown of Prx4 provides resistance to tumor formation by reducing inflammation and promoting cell death without affecting cell proliferation. This finding identifies Prx4 as a potential therapeutic target for prevention and treatment of colorectal cancer in the clinic. Regulation of Prx4 expression in patients at high risk of inflammation-driven cancer through dietary supplements or other preventive agents may prove useful to block or delay the early stages of cancer. Similarly, identification of Prx4-specific inhibitors to target increased apoptosis of tumor cells in combination with radiation or chemotherapy could enhance the outcome of cancer treatment.

## Data Availability

The data underlying this article are available in the article and Supplementary Materials.

## Supplementary Materials

The following supporting information can be downloaded at https://www.mdpi.com/article/10.3390/antiox12030677/s1: Figure S1. Gross images of colons extracted from AOM/DSS-treated mice; Figure S2. Bioinformatics analysis of RNA-sequencing dataset using Immgen; Figure S3. Layout of proteome profiler mouse chemokines and cytokines arrays; Figure S4. Prx4 knockout colons have lower immune cell infiltration compared to wildtype after DSS treatment.

## Abbreviations

AOM/DSS	azoxymethane/dextran sulfate sodium
CRC	colorectal cancer
ER	endoplasmic reticulum
IHC	immunohistochemistry
PD-1	programmed cell death protein 1
Prx	peroxiredoxin
ROS	reactive oxygen species
Srx	sulfiredoxin
TUNEL	terminal deoxynucleotidyl transferase dUTP nick end labeling
