# Supplementary material for: Loss of Peroxiredoxin IV Protects Mice from Azoxymethane/Dextran Sulfate Sodium-Induced Colorectal Cancer Development
Source: Antioxidants (Basel). 2023 Mar 9;12(3):677. doi: 10.3390/antiox12030677 (PMC10045277; doi:10.3390/antiox12030677)
Supplement: Supplementary file 1 [file antioxidants-12-00677-s001.zip › antioxidants-2228269-supplementary.pdf]

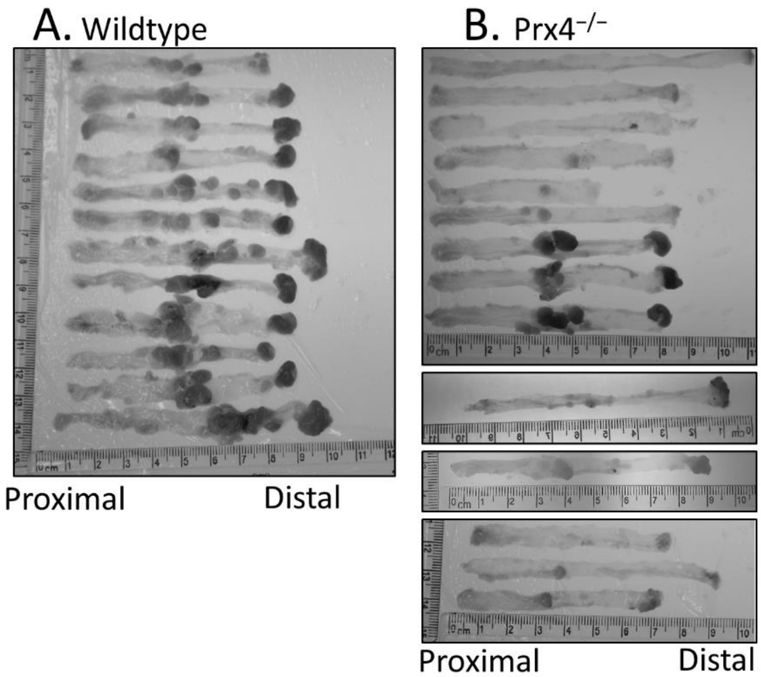

Figure S1. Gross images of colons extracted from AOM/DSS treated (A) wildtype and (B) Prx4 knockout mice.

A.

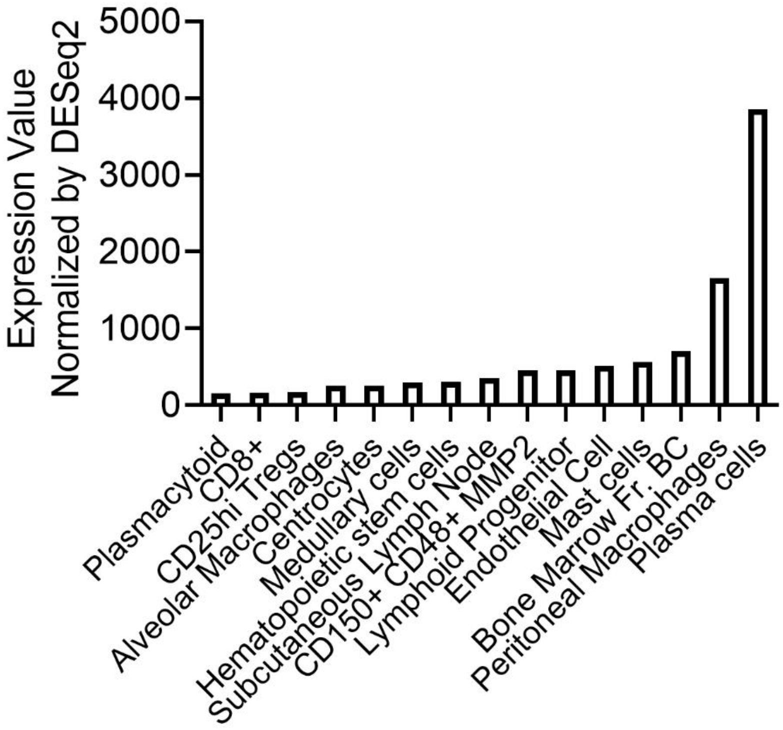

Population Full Name	Population Sorting Description
Splenic Plasmacytoid	sorted on CD45lo CD11b+
Spleenic CD8+	sorted on CD45+ MHCII+ CD11c+ CD8+ CD4-
Splenic CD25hi Tregs	sorted on CD4+CD8-TCRbhiCD62LhiCD44loCD25hiDump-
Alveolar Macrophages	sorted on CD45+ CD11c+ SiglecF+
Splenic Germinal Center Centrocytes	sorted on CD19+B220+IgD-Fas+CD38-CXCR4lo CD86hi
Thymic Medullary Epithelial cells, MHCIIhi	sorted on CD45-Ly51loMHC-IIhiEPCAMhi
Bone Marrow 34- LTHSC/Bone Marrow 34- Long Term hematopoietic stem cells	sorted on Lin-Sca1+ckit+CD135-CD150+CD48-CD34-
Subcutaneous Lymph Node	sorted on CD45- CD31- PDPN- ITGA7+
Bone Marrow CD150+ CD48+ MMP2	
Bone Marrow Common Lymphoid Progenitor	sorted on LIN-CD93+CD117+IL7Ra+CD45R-
Subcutaneous Lymph Node Lymphatic Endothelial Cell	sorted on CD45- CD31+ PDPN+
Peritoneal Mast cells	
Bone Marrow Fr. BC (Pro-B)	sorted on CD93+IgM-CD19+CD43+HSA+
Peritoneal Macrophages	sorted on F4/80+ICAM2+CD5-CD19-CD43-
Splenic Plasma cells	6 week old C57BL/6J BLIMP1-GFP mice sorted on GFPhi CD138+ MHCIIlo (dump: NK1.1- TCRb- Gr1- CD11b-)

Figure S2. (A) Bioinformatics analysis of RNA sequencing dataset using Immgen shows high expression of Prx4 in peritoneal macrophages and splenic plasma cells of 6 week old C57BL/6J mice.

A.

	1	2	3	4	5	6	7	8	9	10	11	12	13	14	15	16	17	18	19	20
A	Reference																		Reference	
B			6Ckine		BLC		C10		C5/C5a		CCL28		Chemerin		CTACK		CXCL16			
C			Eotaxin		Fractalkine		IL-16		IP-10		I-TAC		JE		KC		LIX			
D			MCP-2		MCP-5		MDC		MIG		MIP-1α/β		MIP-1γ		MIP-2		RANTES			
E			SDF-1		Adipsin		Gp130		HSP60		Control (-)									
F	Reference																			

B.

	1	2	3	4	5	6	7	8	9	10	11	12	13	14	15	16	17	18	19	20	21	22	23	24
A	Reference		Acrp30		Amphiregulin		Angpt1		Angpt2		Angptl3		BAFF		C1q R1		CCL2		CCL3/CCL4		CCL5		Reference	
B			CCL6		CCL11		CCL12		CCL17		CCL19		CCL20		CCL21		CCL22		CD14		CD40			
C			CD160		Chemerin		Chitinase 3-like 1		CD142		C5/C5a		Complement Factor D		CRP		CX3CL1		CXCL1		CXCL2			
D	CXCL9		CXCL10		CXCL11		CXCL13		CXCL16		Cystatin C		DKK-1		CD26		EGF		Endoglin		Endostatin		Fetuin A	
E	FGF acidic		FGF-21		Flt-3 Ligand		Gas 6		G-CSF		GDF-15		GM-CSF		HGF		ICAM-1/CD54		IFN-γ		IGFBP-1		IGFBP-2	
F	IGFBP-3		IGFBP-5		IGFBP-6		IL-1α/IL-1F1		IL-1β/IL-1F2		IL-1ra/IL-1F3		IL-2		IL-3		IL-4		IL-5		IL-6		IL-7	
G	IL-10		IL-11		IL-12 p40		IL-13		IL-15		IL-17A		IL-22		IL-23		IL-27 p28		IL-28A/B		IL-33		LDL R	
H	Leptin		LIF		Lipocalin-2		LIX		M-CSF		MMP-2		MMP-3		MMP-9		Myeloperoxidase		Osteopontin		TNFRSF11B		PD-ECGF	
I	PDGF-BB		Pentraxin 2		Pentraxin 3		Periostin		Pref-1		Proliferin		PCSK9		RAGE		RBP4		Reg3G		Resistin			
J	Reference		E-Selectin		P-Selectin		Serpin E1		Serpin F1		Thrombopoietin		TIM-1		TNF-α		VCAM-1		VEGF		WISP-1		Control (-)	

Figure S3. Layout of Proteome profiler mouse chemokine (A) and cytokine (B) arrays adapted from manufacturer's datasheet.

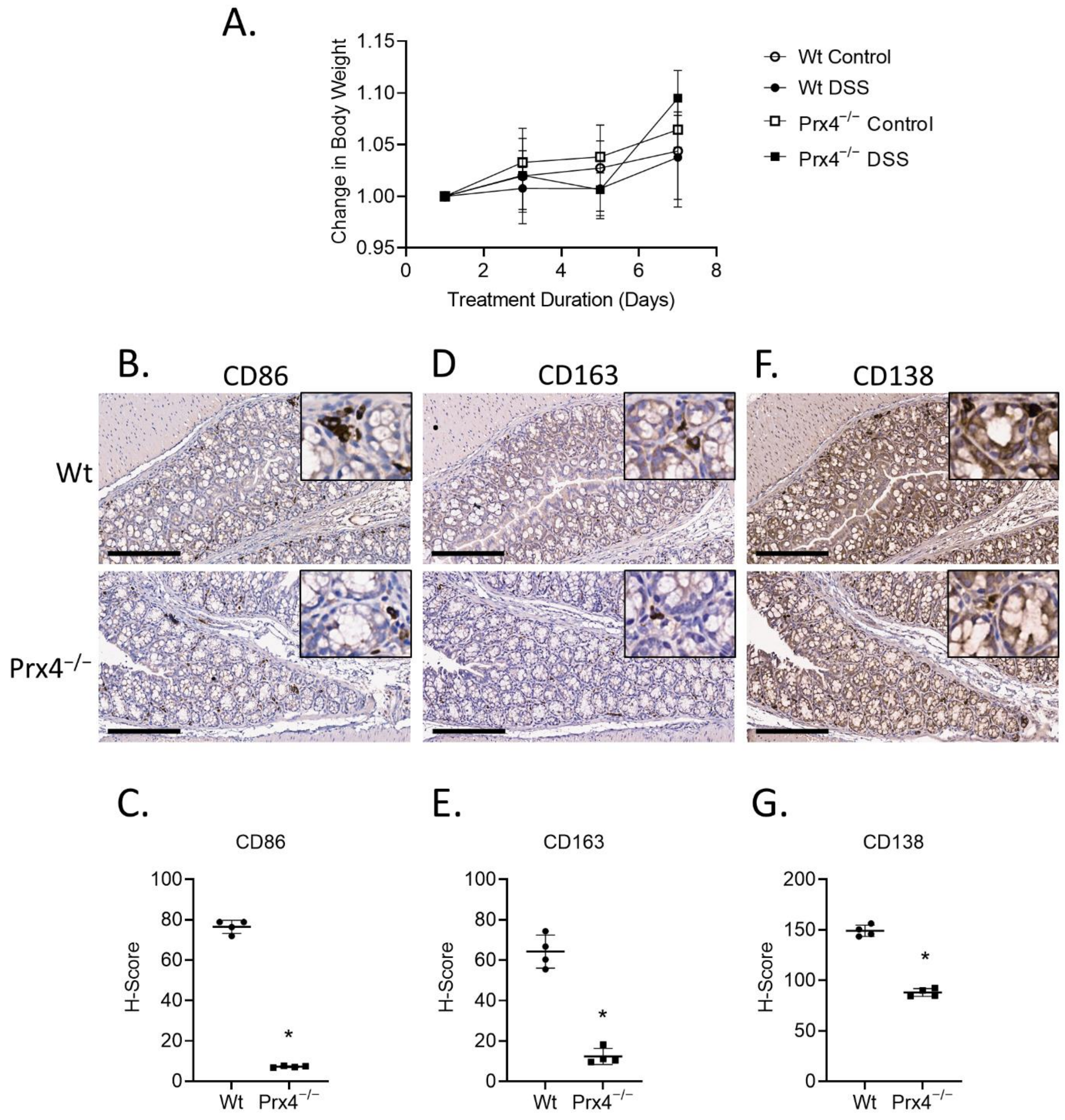
A.
Change in Body Weight
1.15
1.10
1.05
1.00
0.95
0 2 4 6 8
Treatment Duration (Days)
Wt Control
Wt DSS
Prx4−/− Control
Prx4−/− DSS
B.
CD86
D.
CD163
F.
CD138
Wt
Prx4−/−
C.
CD86
H-Score
100
80
60
40
20
0
Wt
Prx4−/−
*
E.
CD163
H-Score
100
80
60
40
20
0
Wt
Prx4−/−
*
G.
CD138
H-Score
200
150
100
50
0
Wt
Prx4−/−
*

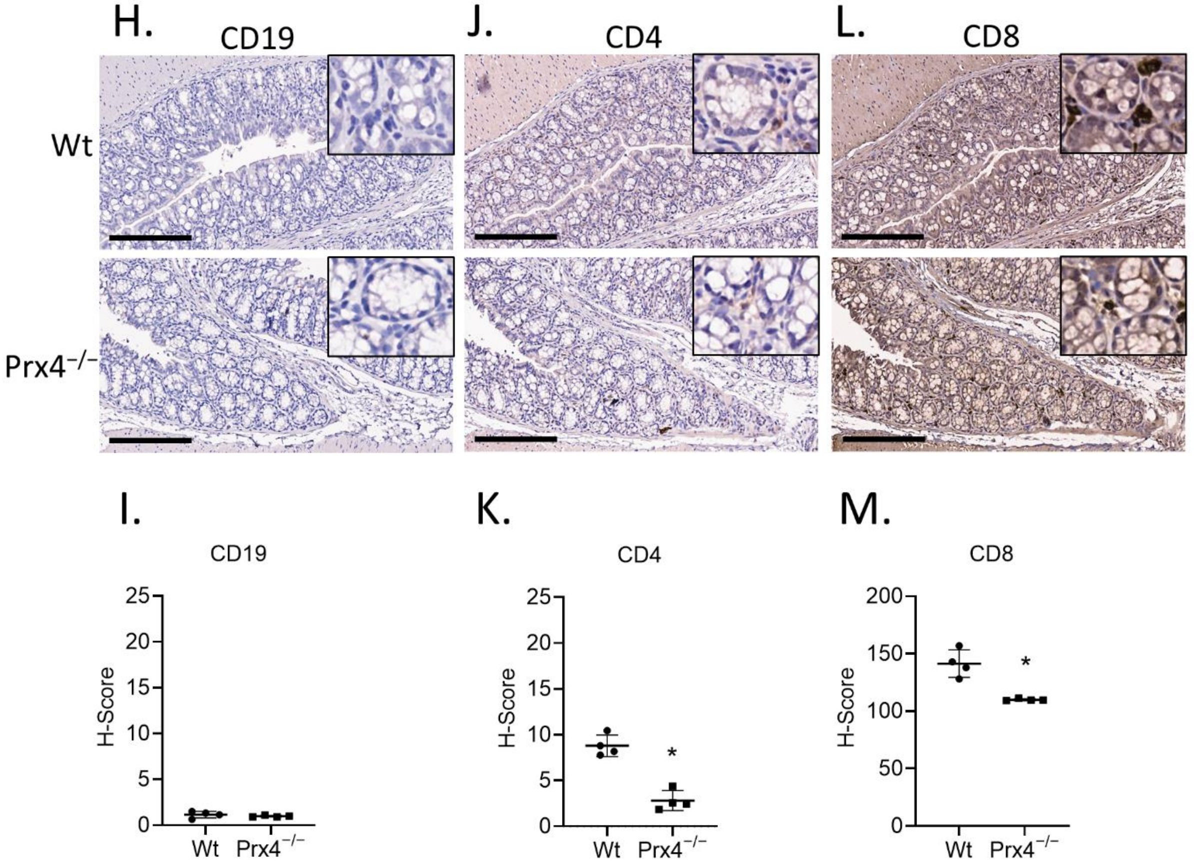

Figure S4. Prx4 knockout colons have lower immune cell infiltration compared to wildtype after DSS treatment. (A) Change to initial body weight of DSS-treated or untreated (control) wildtype and Prx4 knockout mice. Decreased macrophage infiltration in Prx4 knockout colon as indicated by staining of M1 macrophage marker CD86 (B and C) and M2 macrophage marker CD163 (D and E). (F and G) IHC staining of CD138 indicates lower presence of plasma cells in Prx4 knockout colon. (H and I) Comparable naïve B cells infiltration as indicated by staining of CD19. (J and K) Lower levels of CD4+ staining was detected in Prx4 knockout compared to Wt. (L and M) IHC staining of CD8 shows decreased recruitment of cytotoxic T cells in Prx4KO group. Compared with Wt group, *p<0.05 (Student's t-test). Bar = 200 μm
